# Head Injuries and Serial Killers: Explore the Link Between Head Trauma and Criminal Behaviour

**DOI:** 10.1192/bjo.2025.10206

**Published:** 2025-06-20

**Authors:** Melissa Nwako

**Affiliations:** Trakia Medical University, Stara Zagora, Bulgaria

## Abstract

**Aims:** This research aims to investigate whether there is a significant correlation between head injuries and the development of violent, repetitive criminal behaviours, particularly serial killers. Examining the neurological and psychological factors associated with head injuries. This study seeks to understand better their influence on criminal tendencies and patterns of behaviour.

**Methods:** 1. Neuroimaging: This showed reduced amygdala and frontal cortex interconnection.

2. Documented cases of serial killers with a history of head injuries.

3. Nature and timing of head injury; behavioural changes post-injury.

4. Statistics from findings out of 11 serial killers that were studied.

**Results:** 1. Neuroimaging showed reduced amygdala and frontal cortex interconnection and decreased grey matter.

2. High-profile serial killers who had documented head injuries: Richard Ramirez, Glen Edward Rogers, and John Wayne Gacy. Arthur Shawcross, Fred West.

3. Nature: Richard Ramirez, aged 2; a dresser fell on him and aged 5 was knocked out by a swing in the park; both of these caused him to have epileptic seizures throughout his childhood (temporal lobe epilepsy). Glen Edward Rogers, aged 1–2, would rock back and forth, continually banging his forehead against hard surfaces; Arthur Shawcross, aged 16, was hit in the head with a sports discus; and Arthur Shawcross, aged 19, fell off a ladder, concussing himself. Fred West, aged 17, had a motorcycle accident, and aged 19, was punched in the face, which led him to fall two floors, causing him to black out and frequently suffer from violent rages. Brain injuries before the age of 5 permanently disrupt the development of key foundational brain structures, whereas brain injuries in teenage years disrupt ongoing development, altering existing behaviour. Behavioural changes post-injury: emotional instability, social withdrawal, impulsiveness, and poor decision-making.

4. 80% of the most high-profile serial killers have had significant brain injuries.

**Conclusion:** The findings suggest that head injuries, especially those affecting specific brain regions, can lead to problems with impulse control, emotional regulation, and decision-making. Findings also suggest that timing plays a key role too. Early-life brain injuries, particularly during critical developmental stages, disrupt emotional and social development, whereas brain injuries during adolescence often impair impulse control and judgment. For example, the parts of Richard Ramirez’s brain that were damaged were his prefrontal cortex and temporal lobe. These injuries link to his crime as his crimes escalated in brutality, his sadistic behaviour, and also his opportunistic and impulsive nature. Arthur Shawcross similarly, although his injuries were in adolescence, led to sexual deviance and compulsions leading to abnormal sexual behaviour.

